# Supplementary figures and images for: Comparison of qPCR protocols for quantification of “Candidatus Saccharibacteria”, belonging to the Candidate Phyla Radiation, suggests that 23S rRNA is a better target than 16S rRNA
Source: PLoS One. 2024 Dec 26;19(12):e0310675. doi: 10.1371/journal.pone.0310675 (PMC11670941; doi:10.1371/journal.pone.0310675)

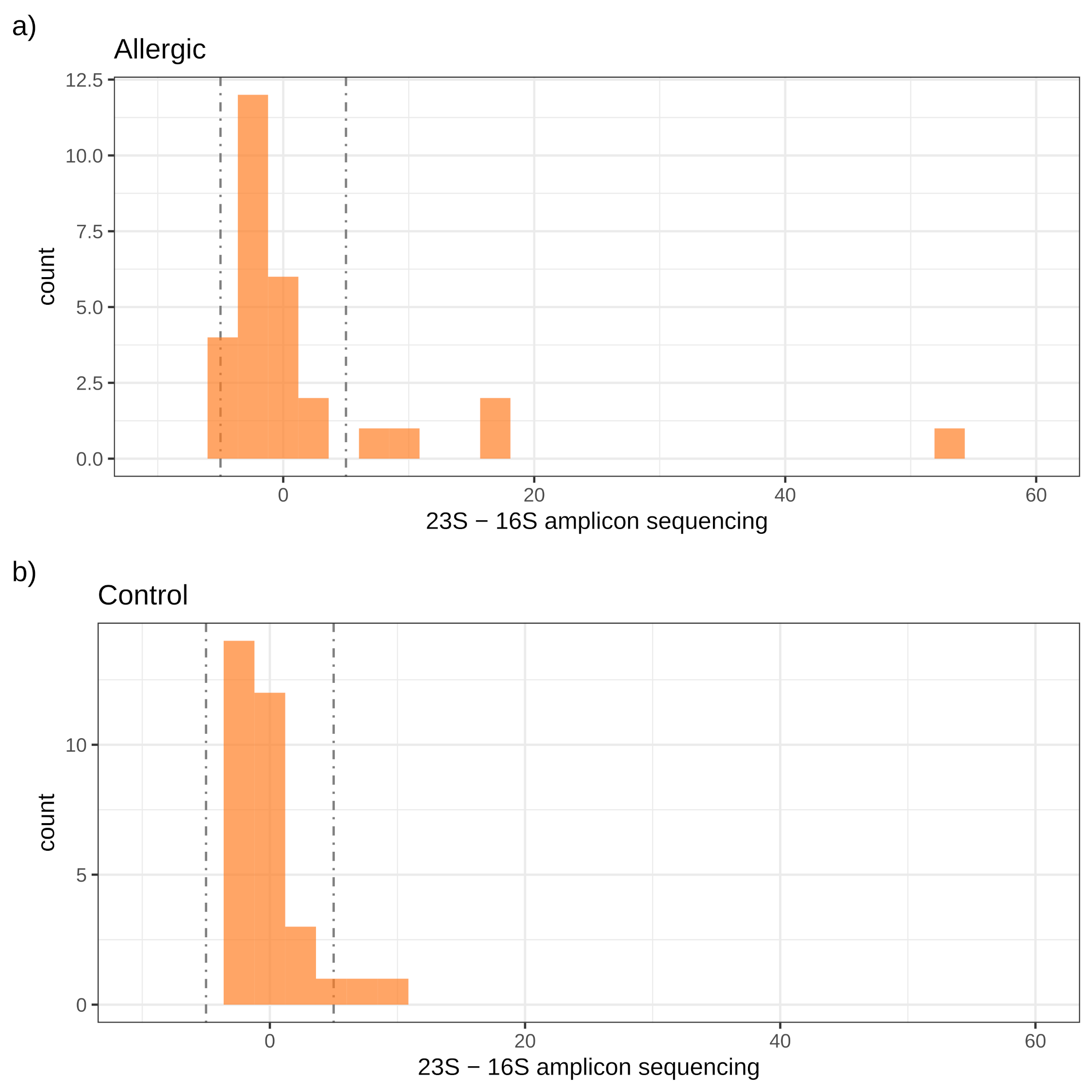

Supplement: S1 Fig — The two histograms report the distribution of the differences between the “Candidatus Saccharibacteria” quantifications obtained by 23S qPCR and by V3-V4 16S rRNA sequencing. Top: distribution of the differences for the allergic group. Bottom: distribution for controls. Dashed vertical lines indicate the interval between -5% and +5% difference between the 16S sequencing and 23S qPCR estimates. (TIF) [file pone.0310675.s001.tif]

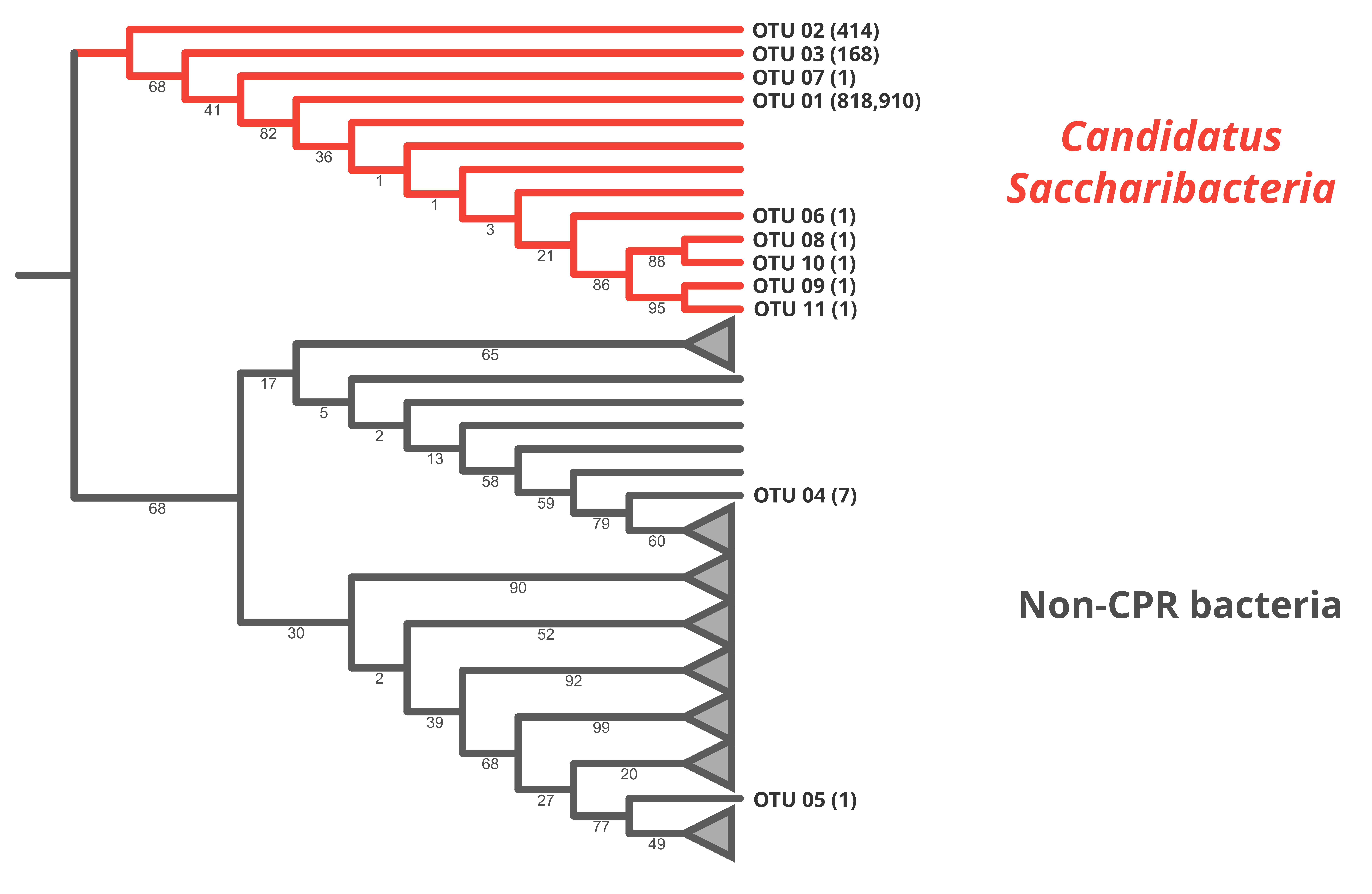

Supplement: S2 Fig — ML tree of the sequences representative of the OTUs obtained from the 23S amplicons and of background sequences retrieved from the nt NCBI database after BlastN search. “Candidatus Saccharibacteria” clade is coloured in red and the clade including non-CPR sequences is in gray. The number of sequences included in each OTU is reported on the leaves. Labels of the leaves from sequences retrieved from the NCBI nt database are omitted. (TIF) [file pone.0310675.s002.tif]
