# Supplementary material for: Comparison of qPCR protocols for quantification of “Candidatus Saccharibacteria”, belonging to the Candidate Phyla Radiation, suggests that 23S rRNA is a better target than 16S rRNA
Source: PLoS One. 2024 Dec 26;19(12):e0310675. doi: 10.1371/journal.pone.0310675 (PMC11670941; doi:10.1371/journal.pone.0310675)
Supplement: S1 Table — List of the primer pairs tested in this work. (DOCX) [file pone.0310675.s003.docx]

**S1 Table.** List of the primer pairs tested in this work.

| **Target group** | **Primer name** | **Sequence (5′–3′)** | **Protocol name** | **Amplicon length (bp)** | **Reference** |
| --- | --- | --- | --- | --- | --- |
| *Ca. Saccharibacteria* | SacchariF | GGCTTATAGCGCCCAATAG | 23S | 126 | [Ibrahim et al. 2021](https://paperpile.com/c/qCgTEd/veI6) |
| *Ca. Saccharibacteria* | SacchariR | CGGATATAAACCGAACTGTC | 23S | 126 | [Ibrahim et al. 2021](https://paperpile.com/c/qCgTEd/veI6) |
| *Ca. Saccharibacteria* | TM7314F | GAGAGGATGATCAGCCAG | 16S_p1 | 596 | [Hugenholtz et al. 2001](https://paperpile.com/c/qCgTEd/kxdK) |
| *Ca. Saccharibacteria* | TM7-910R | GTCCCCGTCAATTCCTTTATG | 16S_p1 | 596 | [Brinig et al. 2003](https://paperpile.com/c/qCgTEd/BFie) |
| *Ca. Saccharibacteria* | Sac1031F | AAGAGAACTGTGCCTTCGG | 16S_p2 | 187 | [Yang et al. 2015](https://paperpile.com/c/qCgTEd/gJbM) |
| *Ca. Saccharibacteria* | Sac1218R | GCGTAAGGGAAATACTGACC | 16S_p2 | 187 | [Yang et al. 2015](https://paperpile.com/c/qCgTEd/gJbM) |
| *Ca. Saccharibacteria* | TM7_16S_590F | GWAAAGAGTWGCGTAGGYGG | 16S_p3 | 375 | [Ferrari et al. 2014](https://paperpile.com/c/qCgTEd/yLCH) |
| *Ca. Saccharibacteria* | TM7_16S_965R | WTRCTTAACGCGTTAGCTTCGCT | 16S_p3 | 375 | [Ferrari et al. 2014](https://paperpile.com/c/qCgTEd/yLCH) |
| Universal | 926F | AAACTCAAAKGAATTGACGG | 16S_panbacteria | 136 | [Bacchetti De Gregoris et al. 2011](https://paperpile.com/c/qCgTEd/8NUB) |
| Universal | 1062R | CTCACRRCACGAGCTGAC | 16S_panbacteria | 136 | [Bacchetti De Gregoris et al. 2011](https://paperpile.com/c/qCgTEd/8NUB) |
| Metagenomics | pro314F | CCTACGGGNBGCASCAG | 16S_meta | 491 | Takahashi et al 2014 |
| Metagenomics | pro805R | GACTACNVGGGTATCTAATCC | 16S_meta | 491 | Takahashi et al 2014 |

**References**

[Bacchetti De Gregoris, Tristano, Nick Aldred, Anthony S. Clare, and J. Grant Burgess. 2011. “Improvement of Phylum- and Class-Specific Primers for Real-Time PCR Quantification of Bacterial Taxa.” *Journal of Microbiological Methods* 86 (3): 351–56.](http://paperpile.com/b/qCgTEd/8NUB)

[Brinig, Mary M., Paul W. Lepp, Cleber C. Ouverney, Gary C. Armitage, and David A. Relman. 2003. “Prevalence of Bacteria of Division TM7 in Human Subgingival Plaque and Their Association with Disease.” *Applied and Environmental Microbiology* 69 (3): 1687–94.](http://paperpile.com/b/qCgTEd/BFie)

[Ferrari, Belinda, Tristrom Winsley, Mukan Ji, and Brett Neilan. 2014. “Insights into the Distribution and Abundance of the Ubiquitous Candidatus Saccharibacteria Phylum Following Tag Pyrosequencing.” *Scientific Reports* 4 (February): 3957.](http://paperpile.com/b/qCgTEd/yLCH)

[Hugenholtz, P., G. W. Tyson, R. I. Webb, A. M. Wagner, and L. L. Blackall. 2001. “Investigation of Candidate Division TM7, a Recently Recognized Major Lineage of the Domain Bacteria with No Known Pure-Culture Representatives.” *Applied and Environmental Microbiology* 67 (1): 411–19.](http://paperpile.com/b/qCgTEd/kxdK)

[Ibrahim, Ahmad, Mohamad Maatouk, Andriamiharimamy Rajaonison, Rita Zgheib, Gabriel Haddad, Jacques Bou Khalil, Didier Raoult, and Fadi Bittar. 2021. “Adapted Protocol for Cocultivation: Two New Members Join the Club of Candidate Phyla Radiation.” *Microbiology Spectrum* 9 (3): e0106921.](http://paperpile.com/b/qCgTEd/veI6)

[Yang, Yun-Wen, Mang-Kun Chen, Bing-Ya Yang, Xian-Jie Huang, Xue-Rui Zhang, Liang-Qiang He, Jing Zhang, and Zi-Chun Hua. 2015. “Use of 16S rRNA Gene-Targeted Group-Specific Primers for Real-Time PCR Analysis of Predominant Bacteria in Mouse Feces.” *Applied and Environmental Microbiology* 81 (19): 6749–56.](http://paperpile.com/b/qCgTEd/gJbM)
