# Supplementary material for: Comparison of qPCR protocols for quantification of “Candidatus Saccharibacteria”, belonging to the Candidate Phyla Radiation, suggests that 23S rRNA is a better target than 16S rRNA
Source: PLoS One. 2024 Dec 26;19(12):e0310675. doi: 10.1371/journal.pone.0310675 (PMC11670941; doi:10.1371/journal.pone.0310675)
Supplement: S2 Table — Thermal profiles used in the PCR experiments. (DOCX) [file pone.0310675.s004.docx]

**S2 Table.** Thermal profiles used in the PCR experiments.

| **Primer set PCR** | **Protocol Name** | **Thermal Profile** |
| --- | --- | --- |
| SacchariF-SacchariR | 23S | 95 °C for 3 min  [95 °C for 30 s  60 °C for 30 s  72 °C for 30 s] x33  75 °C for 5 min |
| 16S_TM7_314F_910R | 16S p1 | 95 °C for 3 min  [95 °C for 30 s  62 °C for 30 s  72 °C for 30 s] x33  75 °C for 5 min |
| 16S_TM7_1031F_1218R | 16S p2 | 95 °C for 5 min  [95 °C for 15 s  61.5°C for 15 s  72 °C for 20 s] x39  75 °C for 5 min |
| 16S_TM7_590F_965R | 16S p3 | 94 °C for 5 min  [94 °C for 30 s  61 °C for 30 s  72 °C for 30 s] x34  72 °C for 5 min |
| 16S_univ_926F_1062R | 16S_panbacteria | 95 °C for 5 min  [95 °C for 15 s  61.5°C for 15 s  72 °C for 20 s] x39  75 °C for 5 min |
