# Supplementary material for: Comparison of qPCR protocols for quantification of “Candidatus Saccharibacteria”, belonging to the Candidate Phyla Radiation, suggests that 23S rRNA is a better target than 16S rRNA
Source: PLoS One. 2024 Dec 26;19(12):e0310675. doi: 10.1371/journal.pone.0310675 (PMC11670941; doi:10.1371/journal.pone.0310675)
Supplement: Attachment — Submitted filename: response to reviewers (1).docx [file pone.0310675.s010.docx]

**JOURNAL REQUIREMENTS:**

Journal Requirements:

Please review your reference list to ensure that it is complete and correct. If you have cited papers that have been retracted, please include the rationale for doing so in the manuscript text, or remove these references and replace them with relevant current references. Any changes to the reference list should be mentioned in the rebuttal letter that accompanies your revised manuscript. If you need to cite a retracted article, indicate the article’s retracted status in the References list and also include a citation and full reference for the retraction notice.

Done

**Comments to the Author**

1. If the authors have adequately addressed your comments raised in a previous round of review and you feel that this manuscript is now acceptable for publication, you may indicate that here to bypass the “Comments to the Author” section, enter your conflict of interest statement in the “Confidential to Editor” section, and submit your "Accept" recommendation.

Reviewer #1: All comments have been addressed

Reviewer #2: All comments have been addressed

2. Is the manuscript technically sound, and do the data support the conclusions?

The manuscript must describe a technically sound piece of scientific research with data that supports the conclusions. Experiments must have been conducted rigorously, with appropriate controls, replication, and sample sizes. The conclusions must be drawn appropriately based on the data presented.

Reviewer #1: Partly

Reviewer #2: Yes

3. Has the statistical analysis been performed appropriately and rigorously?

Reviewer #1: I Don't Know

Reviewer #2: Yes

4. Have the authors made all data underlying the findings in their manuscript fully available?

Reviewer #1: Yes

Reviewer #2: Yes

5. Is the manuscript presented in an intelligible fashion and written in standard English?

Reviewer #1: Yes

Reviewer #2: Yes

6. Review Comments to the Author

Reviewer #1: Authors have responded to all my comments, but refuse to address and do follow up experiment with any of them. They pointed out that they ran out of sample DNA, and recommended comments were too much work for current manuscript. If this article is only repeating what was done before, I am not sure what it is adding to the field. Improving the identification of Saccharibacteria using new qPCR primers will achieve such results.

We assume that this comment refers to point 3 of the original revision, because this is the only point where, in our response, we stated that it was not possible for us to proceed with further experiments with this dataset of DNAs. These samples, in fact, were used for previous work and, physically, many of them are now finished.

In our answer to this point, we explain this. We also briefly discuss why we used the V3-V4 rRNA amplicon sequencing instead of the V1-V3 mentioned by the reviewer. We explain how, in light of recent bioinformatics analysis (for which we cite the relative papers), the V3-V4 16S rRNA amplicon sequencing (and specifically the primer pair we used) performs very well in the context of oral microbiota because it allows optimal OTU clustering at the 97% level, avoiding clustering together sequences belonging to distinct genera. This is a problem that frequently affects several of the most frequently used primers pairs for the description of the oral microbiota.

Moreover, to further address the point raised by the reviewer, in our first revision we added a sentence in the Conclusions, highlighting that our results were obtained by targeting the V3-V4 regions, but that we could not exclude differences in the results if targeting other V regions.

As explained above, we are not in a position to carry out further experiments. However, to further address the point raised by the referee we now specify in the abstract that our results are based on the V3-V4 regions of the 16S rRNA.

We would also like to add that we disagree with the reviewer, when he/she states that the paper is “only repeating what was done before, I am not sure what it is adding to the field”. This is not the first paper to compare methods, both experimental and bioinformatic. Unlike the reviewer, we have always thought that these are very useful analyses for the community of scientists working on a specific topic. This applies even more to a field such as that of candidate phyla radiation (CPR) and its role in the human microbiota. Research in this field is still in its infancy and with a strong need of understanding “what” the current protocols are able to “see”, both in terms of “who is in there” and “how many”. Naturally, the need for new, more focussed protocols is a pivotal point, to be addressed rigorously, with dedicated work.

Reviewer #2: I compared the authors' responses to the comments and am satisfied with the corrections they made to the text of the manuscript.

7. PLOS authors have the option to publish the peer review history of their article ([what does this mean?](https://eur02.safelinks.protection.outlook.com/?url=https%3A%2F%2Fjournals.plos.org%2Fplosone%2Fs%2Feditorial-and-peer-review-process%23loc-peer-review-history&data=05%7C02%7Csimona.panelli1%40unimi.it%7C29573745e1ae412a909508dcb6510790%7C13b55eef70184674a3d7cc0db06d545c%7C0%7C0%7C638585706349165027%7CUnknown%7CTWFpbGZsb3d8eyJWIjoiMC4wLjAwMDAiLCJQIjoiV2luMzIiLCJBTiI6Ik1haWwiLCJXVCI6Mn0%3D%7C0%7C%7C%7C&sdata=j5UkrQGOtQr8KU54NAMSLBsf9BjOzOsDCIHF8w06XHM%3D&reserved=0)). If published, this will include your full peer review and any attached files.

**Do you want your identity to be public for this peer review?** For information about this choice, including consent withdrawal, please see our [Privacy Policy](https://eur02.safelinks.protection.outlook.com/?url=https%3A%2F%2Fwww.plos.org%2Fprivacy-policy&data=05%7C02%7Csimona.panelli1%40unimi.it%7C29573745e1ae412a909508dcb6510790%7C13b55eef70184674a3d7cc0db06d545c%7C0%7C0%7C638585706349171431%7CUnknown%7CTWFpbGZsb3d8eyJWIjoiMC4wLjAwMDAiLCJQIjoiV2luMzIiLCJBTiI6Ik1haWwiLCJXVCI6Mn0%3D%7C0%7C%7C%7C&sdata=KGBPUbZ9SiFzizIASu9lJn4kl9ENGpI1YLVrR2PM1qc%3D&reserved=0).

Reviewer #1: No

Reviewer #2: No
